# Artificial Intelligence for Pre-operative Diagnosis of Malignant Thyroid Nodules Based on Sonographic Features and Cytology Category

**DOI:** 10.1007/s00268-022-06798-1

**Published:** 2022-11-06

**Authors:** Karishma Jassal, Afsanesh Koohestani, Andrew Kiu, April Strong, Nandhini Ravintharan, Meei Yeung, Simon Grodski, Jonathan W. Serpell, James C. Lee

**Affiliations:** 1 Monash University Endocrine Surgery Unit, The Alfred Hospital, 55 Commercial Road, Melbourne, VIC 3004 Australia; 2 Department of Surgery, Central Clinical School, Monash University, Melbourne, Australia

## Abstract

**Background:**

Current diagnosis and classification of thyroid nodules are susceptible to subjective factors. Despite widespread use of ultrasonography (USG) and fine needle aspiration cytology (FNAC) to assess thyroid nodules, the interpretation of results is nuanced and requires specialist endocrine surgery input. Using readily available pre-operative data, the aims of this study were to develop artificial intelligence (AI) models to classify nodules into likely benign or malignant and to compare the diagnostic performance of the models.

**Methods:**

Patients undergoing surgery for thyroid nodules between 2010 and 2020 were recruited from our institution’s database into training and testing groups. Demographics, serum TSH level, cytology, ultrasonography features and histopathology data were extracted. The training group USG images were re-reviewed by a study radiologist experienced in thyroid USG, who reported the relevant features and supplemented with data extracted from existing reports to reduce sampling bias. Testing group USG features were extracted solely from existing reports to reflect real-life practice of a non-thyroid specialist. We developed four AI models based on classification algorithms (*k*-Nearest Neighbour, Support Vector Machine, Decision Tree, Naïve Bayes) and evaluated their diagnostic performance of thyroid malignancy.

**Results:**

In the training group (*n* = 857), 75% were female and 27% of cases were malignant. The testing group (*n* = 198) consisted of 77% females and 17% malignant cases. Mean age was 54.7 ± 16.2 years for the training group and 50.1 ± 17.4 years for the testing group. Following validation with the testing group, support vector machine classifier was found to perform best in predicting final histopathology with an accuracy of 89%, sensitivity 89%, specificity 83%, F-score 94% and AUROC 0.86.

**Conclusion:**

We have developed a first of its kind, pilot AI model that can accurately predict malignancy in thyroid nodules using USG features, FNAC, demographics and serum TSH. There is potential for a model like this to be used as a decision support tool in under-resourced areas as well as by non-thyroid specialists.

## Introduction

Thyroid nodules are common. Approximately 7% of the adult population have a palpable thyroid nodule and the prevalence of imaging-detected nodules approaches 70% [1, 2]. However, many incidental nodules are not of clinical significance, and only around 5% are malignant [3]. As surgery is the primary treatment, evaluation by a specialist thyroid surgeon to determine extent of surgery is pivotal in the management of patients with malignant or suspicious thyroid nodules. Nevertheless, general practitioners (GP) and general surgeons should have a reliable, yet cost-effective method of discriminating between benign and malignant nodules, to help guide referrals or surveillance.

Ultrasonography (USG) and fine needle aspiration cytology (FNAC) are the most widely used modalities in clinching the thyroid nodule diagnosis [4–8]. Within USG, thyroid nodules are increasingly classified using the American College of Radiology Thyroid Imaging, Reporting and Data System (TI-RADS) which has a reasonably high diagnostic performance [9, 10]. However, the TI-RADS classification is not only labour intensive but also there is inherent user dependency, inter-reader variability and subjectivity. When there is suspicion based on TI-RADS, FNAC is the most effective diagnostic test. Unfortunately, cytology fails to reach a definitive diagnosis in 10–32% of samples and can be prone to sampling errors in large nodules [11–15].

When applied in the appropriate setting, gene expression and genomic sequencing classifiers (GSC) have been shown to be clinically beneficial and effective in reducing diagnostic thyroidectomy. However, its unproven cost-effectiveness and accessibility issues have limited its use outside the USA. Comparable artificial intelligence (AI) algorithms are increasingly used to deliver solutions or aid in decision-making in many healthcare contexts, including image classification of thyroid nodules [16–18]. Most existing models give the user a static output—malignant vs benign—and are purely radiologically driven.

The overall purpose of this pilot study is to address the shortcomings of thyroid nodule diagnostics. We aimed to develop an AI classifier model by incorporating radiology, cytology, biochemistry and demographic data to estimate the probability of malignancy in a nodule. Secondarily, we aimed to determine the diagnostic performance of the models created.

## Materials and methods

Ethical approval was granted by the institution’s review board.

### Study population

**Fig. 1:**
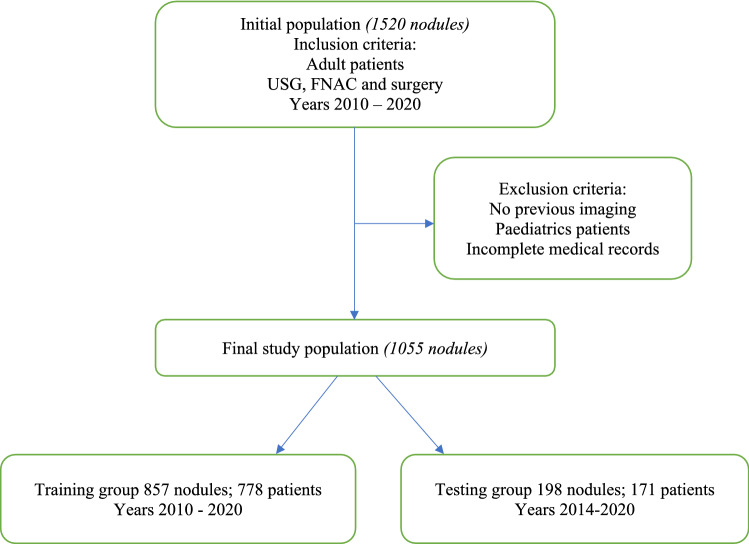
Flow chart of inclusion criteria for training and testing groups

This was a multicentre study from 2010 to 2020. Patients undergoing thyroid surgery were recruited from the prospectively maintained surgical database of the Monash University Endocrine Surgery Unit and assigned to either the training or testing group (approximately an 80/20% distribution). (Fig. 1).

### Ultrasonographic features

The thyroid nodules were assessed for the presence of features commonly used to determine degree of suspicion for malignancy, including solitary nodule, microcalcification, hypoechogenecity, taller-than-wide shape, irregular margins, halo, solid components in a cystic nodule, central vascularity, and associated lymphadenopathy. In the training group, these features were extracted from USG images by a dedicated study radiologist with interest and experience in thyroid imaging in two-thirds of the cases, and from existing USG reports in the remaining cases. This mixed method of extracting features was employed to diversify the training dataset, increase heterogeneity, and reduce sampling bias that can potentially attenuate the performance of the AI model.

To reflect a real-life clinical scenario, the above USG features for the testing group patients were solely extracted from pre-existing reports, without re-interpretation of images. Data extraction was performed by two surgical residents to simulate non-specialist interpretation of radiology vernacular. Discrepancies were addressed and resolved by the senior author (JL). Nodule characteristics not mentioned on the USG reports were considered not present. TI-RADS classification scores were not included; this enabled a pragmatic approach using fundamental USG features and greater flexibility within our model.

### Biochemistry, FNAC and histopathology

In addition to USG features, other clinical parameters collected for inclusion in the machine learning model were age, sex, suppressed serum thyroid-stimulating hormone (TSH) on presentation, and FNAC findings. The presence of suppressed TSH was defined as below the lower limit of the reference range of each laboratory. Cytology findings were reported using the Bethesda system [19].

All included patients had undergone thyroidectomy, and the histopathology was reported using World Health Organisation guidelines [20]. The histological diagnosis was used to label each nodule as benign or malignant. In the training group, this was used to train and internally validate the machine learning algorithm. In the testing group, this was used to determine the performance of the algorithm.

### Classification models

Using the training group data, four classifiers were used to determine the likelihood of malignancy for a particular nodule. We then compared the performance of these four classifiers by applying the testing group data. The premise of classification models is mapping properties of particular examples and assigning data into attribute-value groups. When given a new example, a classifier ascribes it to the best fitted category. The structure of classification models differs from linear discrimination functions to clustering and each classifier has its own attractive properties to the type of dataset it learns from [21]. We therefore selected a variety of commonly used classification models to evaluate their performance on a thyroid dataset.

The selected classifiers were as follows:

1. K-Nearest Neighbour (kNN): Each case is assigned a score, which is calculated using a series of formulae based on examining the entire training cohort. The score of a new case is then compared to the scores of cases in the training group. The new case is then matched to the training case with the closest score, also known as “the nearest neighbour” [21].
2. Decision Tree (DT): The prediction is reached by using a series of branching logic, like a root-to-leaf construct. The order of the branches is determined by the AI after examining the training cohort and determining the relative importance of each parameter [22].
3. Support Vector Machine (SVM): This is thought to be the optimal classifier for determining binary outcomes, such as benignity and malignancy. The theoretical “hyperplane” that separates these 2 outcomes exists in a multi-dimensional space, which consists of as many dimensions as there are the number of parameters [22, 23].
4. Naïve Bayes (NB): Predicts based on Bayes’ theorem with the ‘naïve’ assumption that all parameters are independent given the value of the class variable. [24].

### Statistical analysis and artificial intelligence model

Standard statistical analysis was performed using Stata® software version 17.0 (StataCorp, Texas, USA). Binary variables were analysed using Pearson’s Chi-square test, and continuous variables were analysed using Student’s *t* test. A value of *p* < 0.05 was accepted as statistically significant. The AI model was coded using Python programming language.

To develop the AI model, the above USG features, serum TSH, age, sex and FNAC results were added as parameters and final surgical histology as a target into our models’ data set. Subsequently, a grid search tuning algorithm which is a maximum-likelihood method capable of obtaining optimum results when searching over multi-dimensional spaces, with each parameter considered to add one dimension, was introduced. To train and internally validate our predictive model as well as overcome dataset biases, a resampling technique known as k fold cross-validation was employed [23]. This technique randomly partitions the training group into k fold subsamples (*k* = 10 in this case). *k*-minus-onefold (90%) of the total training group was used as the training subsample and the remaining *k* fold (10%) was used for internal validation within the training group. The partitioning and training occurred ten times over, with a different *k* fold used for internal validation each time. Five repeats of *k* fold cross-validation were performed to improve the estimate of the mean model performance.

**Fig. 2:**
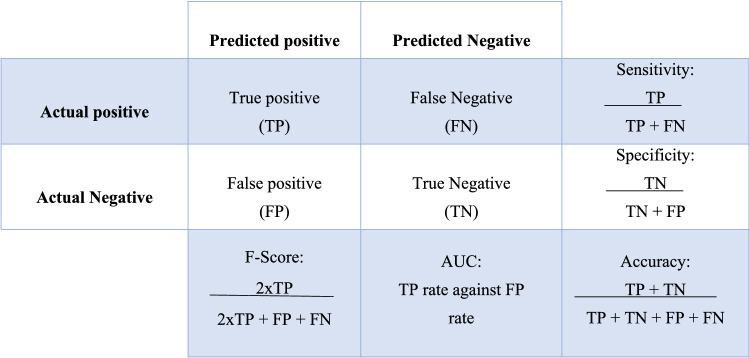
Confusion matrix

The AI predictive model estimates the probability of malignancy in percentage. A value of 50% or greater was accepted as a predicted positive and consequently a true positive if final histology was malignant. Following development and internal validation using the training group, further validation using the testing group was performed for each classifier to determine which had the best performance—measured using a confusion matrix (Fig. 2). Several measures of predictive performance were calculated, including the area under the receiver operating characteristic curve (AUROC), accuracy, sensitivity, specificity, and the F-score. The F-score is a measure of accuracy in binary classification, including both precision and recall [23]. Where numbers were too low to populate the confusion matrix for sub-group analysis, the percentage of correctly classified cases was reported instead.

## Results

The mean age of the study population was 54.7 ± 16.2 years for the training group and 50.1 ± 17.4 years for the testing group (<0.001). After excluding patients with insufficient information, the training group comprised of data of 857 nodules (from 778 patients)—563 re-reported by the study radiologist and 294 had USG features extracted from existing reports. Of these, 624 (73%) cases were benign and 233 (27%) malignant on final histopathology; 641 (75%) patients were female and 216 (25%) males. The testing group included 171 patients with 198 nodules in total. Of these, 164 (83%) were benign and 34 (17%) malignant on final histopathology. There were 153 (77%) female patients and 45 (23%) male patients. Baseline demographics, biochemistry, USG features, cytology and histology findings of the study cohort are summarised in Table 1.

**Table 1:** Demographics of patients and distribution of histopathology, cytology, biochemical and ultrasonographic features of training and testing groups

Features	Training group; *n* = *857*	Testing group; *n* = *198*	*P* value
Mean age, years ± SD	54.7 ± 16.2	50.1 ± 17.4	<0.001
Sex, F (%): M (%)	641 (74.8): 216 (25.2)	153 (77.3): 45 (22.7)	0.47
USG feature extraction, images (%): reports (%)	563 (65.7): 294 (34.3)	0: 198 (100)	-
Histology, benign (%): malignant (%)	624 (72.8): 233 (27.2)	164 (82.8): 34 (17.2)	0.003
Supressed TSH	187	42	0.85
*Cytology*			
Bethesda 1	35 (4.0)	6 (3.0)	<0.0001
Bethesda 2	487 (56.7)	161 (81.3)	
Bethesda 3	125 (14.5)	6 (3.0)	
Bethesda 4	57 (6.6)	10 (5.1)	
Bethesda 5	41 (4.7)	5 (2.5)	
Bethesda 6	117 (13.1)	10 (5.1)	
*Ultrasonography*			
Solitary nodule	299	103	<0.0001
Microcalcifications	125	42	0.003
Lymphadenopathy	35	10	0.54
Hypoechogenecity	128	32	0.67
Taller rather than wide shape	15	3	0.82
Halo	13	5	0.32
Solid – cystic nodule	278	77	0.08
Irregular margins	50	13	0.69
Central vascularity	203	48	0.10

*TSH* Thyroid-stimulating hormone

### Training group results

**Fig. 3:**
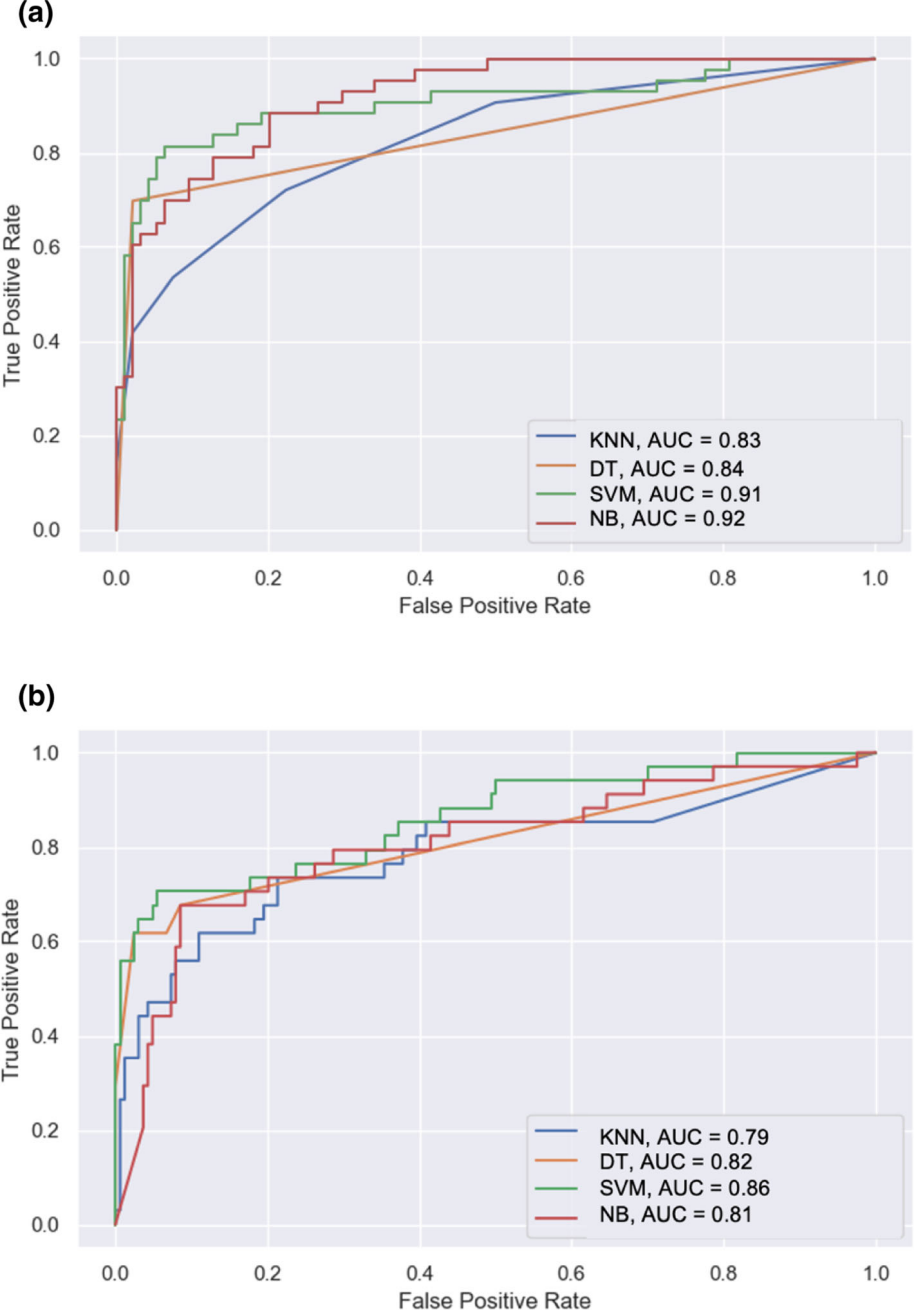
Receiver operating characteristic analysis for the performance of four classifier algorithms tested **a** Training group **b** Testing group

When predictive performance was estimated on the training dataset for each of the four classifiers, SVM performed best with overall accuracy of 89%, sensitivity 81%, specificity 90%, F-score of 86% and AUROC of 0.91. Although DT performed favourably with a slightly higher accuracy and specificity than SVM, it had much lower sensitivity and AUROC. (Table 2a and Fig. 3a).

**Table 2:** Performance analysis of artificial intelligence model

Model	Accuracy (%)	Sensitivity (%)	Specificity (%)	F-Score (%)	AUC (%)
*Performance of classifier models following k fold validation with the training group*					
kNN	76	72	78	75	83
DT	90	70	98	81	84
SVM	89	81	90	86	91
NB	85	74	89	81	92

Model	Accuracy (%)	Sensitivity (%)	Specificity (%)	F-Score (%)	AUC (%)
*Performance of classifier models following validation on the testing group*					
kNN	86	90	60	92	79
DT	87	88	72	92	82
SVM	89	89	83	94	86
NB	79	86	38	87	81

*kNN* K-Nearest neighbour*, DT* Decision tree*, SVM* Support vector machine*, NB* Naïve Bayes

### Testing group results

Similarly, SVM classifier was the best in predicting final histopathology in the testing group, with an accuracy of 89%, sensitivity 89%, specificity 83%, F-score of 94% and AUROC 0.86. It outperformed the other 3 classifiers in all measures, except kNN had a marginally higher sensitivity than SVM (90% vs. 89%), (Table 2b and Fig. 3b).

The SVM classifier correctly predicted 180 of 198 (90.9%) testing group nodules. Of the 18 errors, 15 (7.6%) were false negative predictions and 3 (1.5%) were false positives. Four (26.7%) false negative predictions were incidental micropapillary carcinomas; five (33.3%) had poor quality FNA samples; three (20.0%) were minimally invasive follicular cancers; two (13.3%) papillary cancers in multinodular goitres; and one (6.7%) follicular cancer. The three false positive predictions included two Bethesda 4 nodules incorrectly classified as malignant (one Hurthle cell adenoma and one hyperplastic nodule), and one Bethesda 5 nodule within a multinodular goitre, which was benign histologically.

### Sub-group analysis

We analysed the performance of the classifiers on all six FNAC categories independently within our testing group. For Bethesda 1 and 6 nodules, SVM and DT predicted 100% of final histopathology correctly. With Bethesda 2 nodules, SVM and DT performed similarly at 93.4%. For indeterminate nodules, the percentage of correctly classified nodules for SVM versus DT was 66.1% versus 50.1% for Bethesda 3, 60% versus 70.2% for Bethesda 4, respectively; and for Bethesda 5 nodules, both performed correspondingly classifying 79.9% accurately. *K*NN classified 100% of Bethesda 4 nodules accurately and NB classified 67.7% of Bethesda 3 nodules and 79.9% Bethesda 5 nodules correctly.

### Clinical implications

Within the testing group, there were 16 Bethesda 3 and Bethesda 4 nodules that had diagnostic haemithyroidectomies. There was a high percentage of malignancy within that group, with 9 out of 16 nodules (56.3%) found malignant on operative histology. If the SVM model was applied to this cohort, 5 out of 16 diagnostic haemithyroidectomies (31.3%) that were benign on surgical histology could have been prevented.

There were seven diagnostic haemithyroidectomies performed for Bethesda 4 and Bethesda 5 nodules that the SVM model had predicted as malignant in the testing group. 3 (42.9%) of these patients proceeded to a completion thyroidectomy at a separate admission.

## Discussion

In this study, we designed an AI model to discriminate benign and malignant thyroid nodules based on USG features, FNAC, serum TSH and demographics; trialling four different classifiers. Our model showed high levels of diagnostic performance within the training group with an AUROC of 0.91 for SVM. When further validated on the testing group, SVM also performed best with an AUROC of 0.86; the classifier model had an accuracy of 89% and F-score of 94%. SVM performs well in high dimensional spaces as it creates a hyperplane in a multi-dimensional data space that separates the dataset into two vector sets. When an input element is fed into the SVM system, it is compared in respect to this separating hyperplane [25]. This is likely why SVM performs so well in predicting probability of a binary outcome which in this study’s case is benign versus malignant.

The clinical dilemma that prompted our study lies within two areas. Firstly, in areas with limited access to a specialist endocrine surgical unit, an efficient and cost-effective system to aid interpretation and integration of thyroid nodule diagnostic results would be of high clinical value [26]. Second, generalist surgeons may also benefit from this model. Nonetheless, even in highly specialised units, a diagnostic thyroid lobectomy is often needed to diagnose a nodule with indeterminate cytology [15]. Hypothyroidism post-haemithyroidectomy occurs in 10.9% to 47.0% of patients [27–29]. There is also risk of recurrent laryngeal nerve injury and general operative risks such as bleeding and infection [30, 31]. While the general prevalence of malignancy in indeterminate nodules is around 35–40%, there are series that report rates as low as 6% prompting the need for further risk stratification tools [28, 32, 33].

Most of the recent studies in the field of AI thyroidology have been carried out on computer-aided diagnosis (CAD) systems such as S-Detect (Samsung Medison Co., Seoul, South Korea) which is a real-time classification apparatus incorporated into an ultrasound machine. In these experimental studies, Park et al. and Jeong et al. showed CAD systems had overall comparable diagnostic performance to radiologists with accuracies of 86%. [34, 35] While Chung et al. similarly found that accuracy and sensitivity of the CAD system did not differ from that of a radiologist (88.6% vs. 84.1%, *p* = 0.687; 86.0% vs. 91.0%, *p* = 0.267), the diagnostic performance varied according to the experience level of the USG operator and was lower with less experience [36] Thomas and Haertling [37] developed an image similarity AI tool using convolutional neural network that achieved a sensitivity of 87.8% and specificity of 78.5%. Although images produced by different machines may yield different results, their model allows for the clinician to select the image fed into their model and verify the AI diagnosis by reviewing similar images subsequently to accept or reject the classification of the thyroid nodule provided. This allows the clinician autonomy within the computer support tool and enhances the decision-making process rather than replacing it. Models as such that allow the healthcare practitioner to be involved in multiple steps of the process also allay fears that AI lacking human oversight can result in poor outcomes due to machine error.

In a similar radiologically driven large-scale AI study involving a total of 11,114 patients, Peng et al. [38] found that when their deep learning model assisted radiologists in the diagnostics of a thyroid nodule, the aid of AI improved the AUROC of the performance of radiologists from 0.84 to 0.88 and in their simulated scenario, there was a 26.7% reduction of the need for FNAC and there was a 1.9% decrease in missed malignancies supporting the synergistic relationship between machine and clinician.

While FNAC has been shown to be highly accurate as a screening tool to select patients for surgery or observation, limitations such as insufficient aspirates and results can be susceptible to the challenges of real-world practice especially in areas without specialist interest. Interestingly, a recent meta-analysis suggests that an institution’s malignancy rates influence the interaction between FNAC and USG in indeterminate thyroid nodules where B3 nodules with suspicious USG features from certain centres had a higher probability of malignancy and warranted further action rather than observation [39]. AI appears to be able to provide a potential resolution to these problems by offering a machine-based solution circumventing human modulation. From a clinical perspective, our model works accurately for B1 nodules which could help prevent further aspirates. There are parallel studies in the field of thyroid cytopathology where AI models predict benign vs malignant superiorly compared to FNAC with accuracies up to 95%. Implementation of these models, however, is challenging due to the need for manual segmentation of relevant areas on the cytology slide [40, 41].

In effect, clinically applicable machine learning algorithms in thyroid diagnostics first began with the current commercially available GSC tests that are based on SVM and DT classifiers [42–44]. Advances in molecular markers and genomic sequencing have had positive impacts on individualising treatment for patients with indeterminate cytology. Unfortunately, the availability and feasibility of these advances are currently confined to a few countries. AI models like the one reported in this study could be a possible option for other regions. This AI-driven tool has the potential to improve risk stratification leading to fewer diagnostic lobectomies, better selection of patients for nodule surveillance, and in high-risk cases, enable single-stage surgical planning. It can also be used by GPs to either streamline referrals to a surgical service or empower them to manage benign disease in the community.

By including training data from both the study radiologist and existing reports, we exposed the AI model to a range of reporting styles. The use of existing reports for the testing group further increased the applicability of this model for everyday use. This pilot AI model is the first to incorporate multiple modalities in patient assessment (biochemistry, demographics, radiology, and cytology) into an all-encompassing predictive tool.

There were some limitations to the current study. First, our malignancy rates (25.3%) were lower than some studies (45–52.3%) [45, 46]. However, comparable to other more contemporary studies [37, 47], our study was also susceptible to the limitations of a retrospective design. Additionally, the population of this study was entirely post-operative and does not capture the entire community with thyroid nodules. Addressing this limitation would require a prospective study or retrospective data from patients that are on long-term follow-up for benign or indeterminate nodules that have subsequently proven to be malignant on cytology or continued a benign course. However, this patient population is small, disseminated and to congregate such a cohort to power the AI model to a satisfactory level would require further work and collaboration. Finally, some of the false negative predictions may suggest an over-reliance on the cytology for its predictions, which is likely due to the inclusion of both a high number of Bethesda 2 as well as malignant cytology nodules in the training group. Further work is required to clarify or rectify this point.

Other future work includes improving the model by acquiring a larger dataset and further validating the performance of the model. We are also working on a delivery system that is both easily accessible and user-friendly. The system would be available via a web-based application similar to other online calculators. Once parameters are entered into the system, the user is informed of the probability of malignancy in percentage.

## Conclusion

We have developed a first of its kind pilot AI model that can accurately predict malignancy in thyroid nodules using USG features, FNAC, demographics and serum TSH. Once further evolved and refined for clinical use, there is great potential for this AI model to function as a computer-aided decision support tool, to be used by both surgeons and general practitioners, to help individualise treatment for patients with thyroid nodules.
